# Effect of preheating on the maintenance of body temperature in surgical patients: a randomized clinical trial*

**DOI:** 10.1590/1518-8345.2559.3057

**Published:** 2018-10-25

**Authors:** Cibele Cristina Tramontini Fuganti, Edson Zangiacomi Martinez, Cristina Maria Galvão

**Affiliations:** 1 Universidade Estadual de Londrina, Centro de Ciências da Saúde, Londrina, PR, Brazil.; 2 Universidade de São Paulo, Faculdade de Medicina de Ribeirão Preto, Ribeirão Preto, SP, Brazil.; 3 Universidade de São Paulo, Escola de Enfermagem de Ribeirão Preto, PAHO/WHO Collaborating Centre for Nursing Research Development, Ribeirão Preto, SP, Brazil.

**Keywords:** Hypothermia, Clinical Trial, Perioperative Nursing, Body Temperature, Nursing Research, Health Services

## Abstract

**Objective:**

to evaluate the effect of preheating on the maintenance of body temperature of patients submitted to elective gynecological surgeries.

**Method:**

eighty-six patients were randomized, without blinding, to receive usual care (heating with a cotton sheet and blanket) or preheating with a forced air system for 20 minutes (38°C). All patients were actively heated during the intraoperative period. Data were collected from admission of the patient in the surgical center until the end of the surgery. Body temperature was measured during the preoperative and intraoperative periods with an infrared tympanic thermometer. A thermo-hygrometer was used to monitor air temperature and humidity of the operating room.

**Results:**

data indicated homogeneity between the groups investigated. There was no statistically significant difference between groups after preheating (p = 0.27). At the end of the surgery, the mean temperature of the groups studied was the same (36.8°C), with a statistically non-significant difference (p = 0.66).

**Conclusion:**

preheating with the heated forced air system had a similar effect to the usual care in the body temperature of patients submitted to elective gynecological surgeries. ClinicalTrials.gov n. NCT02422758. CAAE, n. 38320814.2.0000.5393.

## Introduction

The maintenance of the patient’s body temperature during surgery is still a challenge for health professionals despite the advancement of technologies to maintain normothermia and research on the theme. Perioperative hypothermia, defined as core body temperature lower than 36°C, occurs due to the effects of anesthetic drugs, ambient temperature, reduced metabolism, surgical wound extension, and fluid and blood loss. Perioperative hypothermia is a common situation in surgical patients because there are still health services where body temperature is not measured during the surgical anesthetic procedure^1^ ^-^ ^2^.

Complications associated with hypothermia are numerous and even when patients do not develop all of them, they may be susceptible to at least one of them. The most common complications are the occurrence of cardiac events, increased duration of effects of anesthetic drugs, and longer time in the anesthetic recovery room, change in the coagulation cascade, and an increase in the incidence of surgical site infection^(2- 3)^.

Preheating is defined as active heating of peripheral tissues or body surface prior to anesthetic induction^2^. After anesthetic induction, the main cause of hypothermia is the internal redistribution of heat, responsible for approximately 81% of the decrease in body temperature during the first hour of anesthesia, corresponding to a reduction of ca. 1.6°C^4^.

Internal redistribution results from body heat flow from the core (trunk and head) to the peripheral compartments (limbs) and is difficult to be addressed due to the time required to transfer thermal energy from the skin to core compartments^2^ ^,^ ^4^.

Preheating increases the heat content in the peripheral compartment, decreasing the core-periphery temperature gradient, which lessens the redistribution of body heat during the surgical anesthetic procedure^2^ ^,^ ^4^ ^-^ ^5^. The difference between temperature in core and peripheral compartments becomes small, even in adverse conditions, and this can be decisive to maintaining the surgical patient’s body temperature.

It should be emphasized that passive heating, usually obtained with use of cotton bed sheets and blankets, is a conventional method adopted in clinical practice due to lack of resources or lack of knowledge on the part of the health team. There is evidence in the literature that active heating methods are more effective than passive methods to prevent perioperative hypothermia^2^. However, the use of a single layer of passive heating, i.e., the use of a cotton bed sheet can reduce the loss of body heat by around 30%, what may be clinically important^2^.

Research in the literature on preheating is a fruitful theme in the literature, addressing different aspects such as clinical characteristics of patients, type of surgery, type of anesthesia, body temperature measurement technique, intervention choice, duration and moment of preheating^6^ ^-^ ^12^.

Research on preheating has been mostly carried out with adult patients; elderly and children are still poorly studied^1^ ^,^ ^3^ ^,^ ^5^ ^-^ ^16^. The intervention has also been evaluated in different cavity surgical procedures, with open or videolaparoscopic technique^3^ ^,^ ^5^ ^-^ ^15^, and a few studies have analyzed limb surgeries^16^ or regional anesthesia. Regarding the procedure itself, preheating time is not consensus: it ranges from 10 minutes to two hours of intervention^3^ ^,^ ^5^ ^-^ ^16^. Another important point is that there is no information in the studies was about the presence of a time gap between preheating and the beginning of surgery and how this could interfere in the results of the intervention applied.

Although preheating in preoperative surgical patients with the purpose of reducing perioperative hypothermia (reduction of redistribution of heat) are recommended, studies designs that generate strong evidence are scarce in the nursing area and have not been identified so far in Brazilian nursing. Thus, the question of the present research was: “Does preheating for 20 minutes with heated forced air system in elective gynecological surgery patients help in the maintenance of body temperature when compared to usual care (heating with cotton sheet and blanket)?”. The hypothesis of the study is that 20 minutes of preheating with the heated forced air system is able to maintain the body temperature of patients undergoing elective gynecological surgeries when compared to patients who were heated with cotton sheets and blankets.

Perioperative hypothermia is an event that can be prevented^2^ ^,^ ^17^ and nursing plays a fundamental role in the planning of care for surgical patients in all perioperative phases, contributing to the early detection of risks and clinical alterations such as hypothermia^17^, as well as to the creation and implementation of protocols for the management of care, permanent education, insertion of quality indicators of clinical practice, and most importantly improving the outcomes of the care provided.

The maintenance of body temperature in the perioperative period, has an important clinical impact besides the patient comfort, as it can reduce the morbidity associated with hypothermia, reducing bleeding during surgery and incidence of surgical site infection, and consequently hospital stay and health care costs for services^1^ ^-^ ^2^.

The results of the research can guide actions to improve nursing care for surgical patients, as well as increase the knowledge and discussion about perioperative hypothermia.

The purpose of this study was to evaluate the effect of preheating on the maintenance of body temperature in patients submitted to elective gynecological surgeries.

## Method

This is a randomized, non-blinded clinical trial including patients undergoing elective gynecological surgery. Surgeries were carried out at a tertiary private and philanthropic hospital in the north of the state of Paraná from March to October 2015. Eligible patients were aged 18 years or more. The open surgeries lasted at least one hour and the anesthetic technique was general, local or combined. Patients with body temperature below 36°C at admission to the surgical center were excluded. The study was approved by the Research Ethics Committee of the University of São Paulo at Ribeirão Preto College of Nursing under CAAE 38320814.2.0000.5393. The study was also recorded in *ClinicalTrials.gov*, under nº NCT02422758. The consent of all the participants was obtained before their inclusion in the study, while they were in the nursing ward.

For sample calculation, a standard deviation of 0.3 was used for body temperature in both groups based on a pioneer study, considering a difference of 0.2°C between the study groups (clinical significance), with test power of 80% and level of significance of 0.05^13^. The sample size was 37 patients for each group, resulting in a total of 74 patients. Considering the possibility of losses in the study, an increase of 15% was established in the sample, leading to a total of 86 participants, 43 per group.

**Figure 1:**
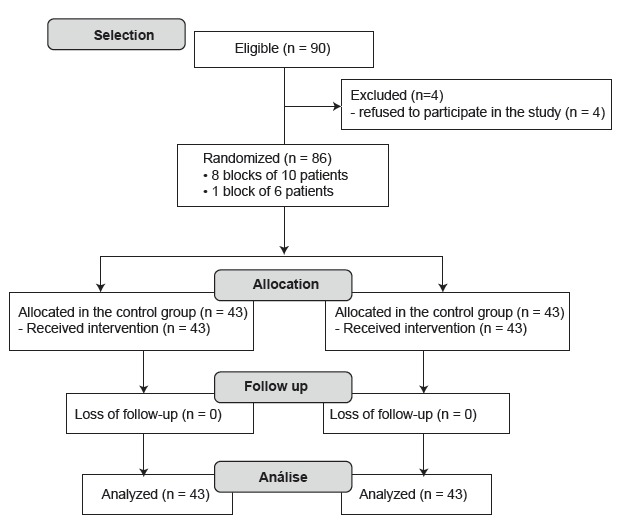
Distribution flowchart of study participants

Patients’ selection and recruitment occurred in the nursing ward of the hospital on the same day of the surgery or one day before the procedure. Participants were randomly assigned to two groups: control and experimental. The randomization procedure was carried out through a list generated by a computer program. The randomization strategy was in blocks, being prepared eight blocks of 10 patients and one of six. The preparation of the list of allocation of participants in the blocks and the preparation of the sealed and opaque envelopes were procedures performed by a person who was not part of the study (Figure 1).

A two-part instrument was developed to achieve the proposed objective. The first part addressed data for patient characterization and identification of factors predisposing to the development of perioperative hypothermia (age, weight and height, sex, American Society of Anesthesiologists Index, ASA, proposed surgery and anesthesia, magnitude of the surgery and associated diseases (heart disease, hypertension, hypothyroidism and Diabetes Mellitus). The second part covered data were related to the implementation of the investigated intervention (preheating), measurement of body temperature and air temperature and humidity of the operating room. The instrument developed in the present study was submitted to face and content validation by five judges (nurses) who evaluated the instrument as having scope and representativeness to reach the objective of the research and also made suggestions regarding the organization of items, formatting, coding and alteration of some terms (for example, instead of Body Mass Index the judges suggested using the record of weight and height, and instead of age, date of birth).

The primary endpoint of the study was the body temperature variation evaluated by means of the tympanic membrane during the surgical anesthetic procedure, before and after preheating (T01 and T02, respectively), at the beginning of the surgery (T03), every thirty minutes during the surgical procedure (T030, T060, T090, T120, T150, T180, T210) and at the end of the procedure (TFINAL). For this purpose an infrared electronic thermometer model GENIUS 2, brand Tyco/Kendall was used. This thermometer measures body temperatures between 33°C and 42°C with accuracy of ± 0.1°C.

Data were collected by one of the authors of the study and occurred as follows: after the patient’s reception in the surgical center, the admission procedure was performed, i.e., checking the identification data and the preoperative preparation, as well as checking of vital signs. At that moment, the body temperature in the tympanic membrane was measured (T01). Then, the sealed and opaque envelope was opened to determine the allocation of the participant. Patients allocated to the experimental group were preheated for 20 minutes with a heated forced air system on medium power (38°C), with a thermal blanket placed over the entire body, while the participants allocated in the control group were covered with cotton bed sheets and blankets (usual care) for 20 minutes. Body temperature was measured after intervention in both groups (T02).

After applying the tested intervention (experimental group), the heated forced air system was shut down and a thermal blanket was kept on the patient until transference to the operating room, and in the control group, the participants remained covered with cotton bed sheets and blankets. All patients waited the moment the surgical room was released for surgery and were, therefore, only passively heated.

After the patient entered the operating room, the tympanic body temperature was measured again. In the operating room before surgery, all participants were covered with a cotton bed sheet and blanket, following the routine of the sector, regardless of the group to which they had been assigned. Venous access and standard monitoring (noninvasive blood pressure, electrocardiographic monitor and pulse oximetry) also followed the routine of the service, as well as the infusion of heated liquids. The air conditioning remained off until the beginning of the surgery, according to hospital routine. The measurement of the patient’s body temperature during the intraoperative period is not an institutional routine.

After anesthetic induction and surgical positioning, the thermal blanket of the heated forced air system was placed on the patients’ upper body of the two groups (experimental and control) and the equipment was turned on at medium power (38°C), remaining this way until the end of the surgery.

Body temperature was measured, always in the same ear, after anesthetic induction, at the beginning of the surgical procedure and consecutively every 30 minutes until the end of the surgery (when the surgical incision suture was completed).

The air temperature and humidity of the operating room were also measured at the patient’s arrival in the room after anesthetic induction at the beginning of the surgical procedure and consecutively every 30 minutes until the end of the surgery. The measurements always occurred close to one meter of the patient’s head and the same side where the tympanic body temperature was measured. For these measurements, we used a Incoterm digital thermo-hygrometer model 7663.02.0.00, with precision of ± 1°C for internal temperature and ± 8% Relative Humidity for ambient humidity.

The independent variable investigated was the preheating of the surgical patient during 20 minutes before anesthetic induction with heated forced air system. This time was determined based on the best practices of the Association of Perioperative Registered Nurses (AORN), in the Guideline for prevention of unplanned perioperative hypothermia (2015)^17^. The dependent variable was the tympanic body temperature.

The database was built through double typing. The quantitative variables age and body mass index were evaluated for measures of position (mean) and dispersion (standard deviation). The variables magnitude of the surgery, type of surgery, type of anesthesia and comorbidities were described by frequency of distribution. The Chi-square test or Fisher’s exact test, and the Mann-Whitney test or Student’s t-test were applied to assess the homogeneity of the groups investigated (experimental and control).

Air temperature and humidity of the operating room and patient waiting time between the end of preheating and entry into the operating room, as well as the variables above mentioned, were analyzed using the Statistical Package for the Social Sciences (SPSS) version 17.0,. The Student’s t-test was used for the comparison of means.

A mixed effect linear regression model was used to compare the participants’ average body temperature between the experimental and control groups at each moment measured. The analyses were conducted in the Statistical Analysis System (SAS) software version 9.3.

For all analyses, the significance level adopted was α = 0.05.

## Results

Clinical characteristics of the patients (Table 1) and surgical anesthetic procedures (Table 2) were compared between groups. The results showed no statistically significant differences.

**Table 1:** Distribution of participants according to clinical characteristics in the control group and the experimental group. Londrina, PR, Brazil, 2015

Characteristics	Control		Experimental		p-value
	(n* = 43)		(n* = 43)		
	Mean	Standard deviation	Mean	Standard deviation	
Age^†^	55.3	13.5	55.6	12.9	0.60^‡^
BMI^§^	29	5.6	27.8	6.5	0.27??
Comorbidities	n*	(%)	n*	(%)	
Gynecological cancer	43	100	43	100	1^¶^
Arterial hypertension	17	39.5	22	51.1	0.38^¶^
Diabetes mellitus	7	16.2	7	16.2	1^¶^
Hypothyroidism	3	6.9	2	4.6	1^¶^
Others	2	4.6	2	4.6	1^¶^

*n-number; †years; ‡p-value resulting from the t-Student test; §Body Mass Index: kg/m^2^; ??p-value resulting from the Mann-Whitney test; ¶p-value resulting from the Chi-square test/Fisher exact test

**Table 2:** Distribution of participants according to characteristics of the surgical anesthetic procedure in the control group and experimental group. Londrina, PR, Brazil, 2015

Characteristics	Control	Experimental	Total	p-value*
	(n^†^=43)	(n^†^=43)	(n^†^=86)	
	n^†^ (%)	n^†^ (%)	n^†^ (%)	
Magnitude of the surgery				1
Magnitude I^‡^ Magnitude II^§^	35 (81.4) 8 (18.6)	34 (79.0) 9 (20.9)	69 (80.2) 17 (19.7)	
Type of surgery				
Hysterectomy	21 (48.8)	25 (58.1)	46 (53.4)	0.51
Gynecological laparotomy	16 (37.2)	15 (34.8)	31 (36.0)	1
Werthein-Meigs hysterectomy	6 (13.9)	3 (6.9)	9 (10.4)	0.48
Type of anesthesia				
Spinal	31 (72.0)	34 (79.0)	65 (75.5)	0.61
Spinal + epidural	1 (2.3)	-	1 (1.1)	1
Spinal + general	3 (6.9)	5 (11.6)	8 (9.3)	0.71
Epidural	2 (4.6)	-	2 (2.3)	0.49
Epidural + general	3 (6.9)	4 (9.3)	7 (8.1)	1
General	3 (6.9	-	3 (3.4)	0.24

*p-value resulting from the Chi-square test/Fisher’s exact test; †n-number; ‡Magnitude I: duration of surgery up to two hours; §Magnitude II: duration of surgery two to four hours

The results of the mixed effect linear regression did not identify a statistically significant difference in the patients’ mean body temperature between the studied groups, at the different moments evaluated during the research. There was difference only between the mean temperatures of the control and experimental groups in the T150 measurement (p = 0.01) (Table 3).

**Table 3:** Distribution of the participants’ mean body temperature before and after preheating until the end of the surgery in the control group and experimental group. Londrina, PR, Brazil, 2015

Time	Control		Experimental		Difference	95% CI*	p-value^†^
	Mean	95% CI*	Mean	95% CI*			
T01^‡^	37.9	(37.7; 38.1)	37.9	(37.8; 38.1)	-0.01	(-0.25; 0.22)	0.91
T02^§^	37.8	(37.7; 38.0)	38.0	(37.8; 38.1)	-0.13	(-0.36; 0.10)	0.27
T03^||^	37.4	(37.3; 37.6)	37.5	(37.3; 37.7)	-0.05	(-0.29; 0.18)	0.66
T030^¶^	37.0	(36.8; 37.2)	37.0	(36.8; 37.2)	-0.01	(-0.24; 0.23)	0.97
T060^**^	36.9	(36.7; 37.1)	36.9	(36.8; 37.1)	-0.07	(-0.32; 0.18)	0.57
T090^††^	36.8	(36.6; 37.0)	36.8	(36.6; 37.0)	0.01	(-0.28; 0.30)	0.97
T120^‡‡^	37.0	(36.8; 37.3)	36.7	(36.5; 37.0)	0.31	(-0.08; 0.70)	0.13
T150^§§^	37.5	(37.1; 37.9)	36.8	(36.4; 37.1)	0.70	(0.15; 1.25)	0.01^||||^
T180^¶¶^	37.4	(36.9; 38.0)	36.9	(36.4; 37.5)	0.52	(-0.27; 1.31)	0.20
T210***	37.4	(36.7; 38.2)	37.2	(36.4; 38.0)	0.26	(-0.84; 1.36)	0.65
Tf^†††^	36.8	(36.7; 37.0)	36.8	(36.6; 37.0)	0.05	(-0.18; 0.29)	0.66

*CI - confidence interval; †p-value resulting from the mixed effect linear regression model; ‡T01 - mean body temperature before preheating; §T02 - mean body temperature after preheating; ||T03 - mean body temperature at the beginning of the surgery; ¶T030 - mean body temperature thirty minutes after the start of the surgery; **T060 - mean body temperature sixty minutes after the start of the surgery; ††T090 - mean body temperature ninety minutes after the start of the surgery; ‡‡T120 - mean body temperature one hundred and twenty minutes after the start of the surgery; §§150 - mean body temperature one hundred and fifty minutes after the start of the surgery; ||||p < 0.05; ¶¶T180 - mean body temperature one hundred and eighty minutes after the start of the surgery; ***T210 - mean body temperature two hundred and ten minutes after the start of the surgery; †††Tf - mean body temperature at the end of the surgery.

After preheating, there was an increase of 0.1°C in the mean body temperature of the patients in the experimental group. As already mentioned, all patients waited for the moment when the operating room was released to start the surgery and, therefore, were only passively heated (passive method, according to the routine of the hospital). This time was of 42.9 minutes on average in the control group (SD = 32.5) and 38.7 minutes in the experimental group (SD = 26.9), with a statistically non-significant difference between groups (p = 0.515).

At the beginning of the surgery, the mean temperature of the operating room was 23.4°C and the mean air humidity was 61.23% in the control group, and 23.6°C and 59.07% in the experimental group. At the end of surgery, the mean temperature of the operating room was 18.9°C and the mean air humidity was 55% in the control group, and 19.5°C and 52% in the experimental group.

The mean temperature of the operating room in the different periods measured was not significantly different between the studied groups. As for air humidity, only in the T120 period the results showed a statistically significant difference between groups (p = 0.03), but this difference did not remain in the moments evaluated afterwards.

## Discussion

Due to the different complications resulting from perioperative hypothermia, the maintenance of body temperature became indicative of the quality standard of patient care provided in the surgical center. Preheating is an intervention that may help reduce perioperative hypothermia^18^. However, in the present study, the results did not show statistically significant differences in the maintenance of body temperature among patients in the experimental group (active heating with the heated forced air system) and in the control group (passive heating).

Clinical trials are found in the literature, and their results are in agreement with the findings of the present study^3^ ^,^ ^7^ ^,^ ^9^ ^,^ ^11^.

In a randomized clinical trial, the authors tested the effectiveness of preheating in 27 patients for a period of 30 minutes, randomized into three groups, namely: no preheating (control group); preheating with heated forced air system (experimental group 1); and preheating with carbon fiber electric cover system (experimental group 2). Both equipment sets were turned on at 42°C. The results indicated the carbon fiber electric cover system as the most effective in maintaining body temperature, and there was no statistically significant difference in the body temperature variation between the experimental group 1 (heated forced air system) and the control group^7^.

In another randomized clinical trial, the effect of preheating was analyzed in 66 patients undergoing colorectal surgery, randomized into two groups. In the control group, the participants were covered with a cotton bed sheet, and in the experimental group, the patients were heated for 30 minutes with heated forced air system. Although the preheating time was planned for 30 minutes, it averaged 75 minutes. The author identified similar proportions of hypothermic patients in both study groups, showing that preheating did not result in less hypothermia among patients^9^.

Participants in a randomized clinical trial (n = 50 elderly patients undergoing transurethral resection surgery) were randomized into two groups: patients not preheated and patients preheated for 20 minutes with heated forced air system (38°C). In both groups there was a decline in body temperature during the intraoperative period (p < 0.001), with a statistically non-significant difference between groups (p = 0.763). The authors concluded that preheating did not prevent perioperative hypothermia but decreased its severity^3^.

Preheating was studied in another clinical trial, but the authors investigated its effect on blood pressure during anesthetic induction. The hypothesis was that preheating would increase the mean of the lowest blood pressure values ​​of patients undergoing neurological surgery during anesthesia compared to non-preheated patients. For one hour, 16 patients were preheated with a heated forced air system (46°C), while another 16 subjects had their bodies covered with thermal blankets, but with the equipment switched off. The results showed that there was no difference in the mean lower ​​blood pressure values (p = 0.36), and hypotension occurred in all patients of the preheated group and in 93% of the patients in the control group. The patients’ core body temperature was higher in the group receiving preheating (p < 0.004), but over time, that temperature changed, with a statistically non-significant difference between groups (p=0.06). The authors concluded that preheating increased core body temperature prior to anesthesia induction, but this did not lead to increased blood pressure or reduced hypotension^11^.

On the other hand there are clinical trials in the literature whose results demonstrated the positive effects of preheating in the reduction of perioperative hypothermia^6^ ^,^ ^8^ ^,^ ^10^ ^,^ ^12^.

The results of a randomized clinical trial showed that preheating with a heated forced air system attenuates hypothermia through redistribution. The sample consisted of 68 adult participants. In the experimental group, patients were preheated with a heated forced air system for 60 minutes (38°C), and compared to the control group (without preheating). All patients were heated with heated forced air system during the intraoperative period. The results showed that the preheated group had a higher core body temperature than the control group (p < 0.005), and patients in the experimental group maintained normothermia more often than patients in the control group (p < 0.05)^6^.

In a clinical trial, the researchers evaluated the action of different preheating times (10, 20 or 30 minutes with heated air system at 44°C) on the prevention of hypothermia and postoperative tremor. The sample consisted of 200 patients randomized into four groups who underwent laparoscopic or open surgery, with a thermal blanket placed on the participants’ upper body of the active preheating groups, and passive heating in the control group. During the intraoperative period, all patients were covered with a cotton bed sheet. The results showed that there was no statistically significant difference between the active preheating groups (p=0.54), but there was a statistically significant difference between the passive preheating group and the three active groups (p < 0.00001)^8^.

In another clinical trial, the authors evaluated the effect of active heating before and/or after epidural anesthesia during general anesthesia (combined anesthesia), and 99 patients undergoing elective abdominal surgery, which lasted at least 120 minutes were randomized into three groups: passive preheating; active preheating 15 minutes after epidural anesthesia;, and preheating active 15 minutes before and 15 minutes after epidural anesthesia. Among patients who received passive preheating (n = 32), 72% had hypothermia at the end of anesthesia, while none of the patients in the pre- and post-preheating group (n = 34) were hypothermic. In the group where active preheating was performed 15 minutes after epidural anesthesia, the incidence of hypothermia was reduced by 6%. The authors concluded that pre-heating the patient 15 minutes before and after epidural anesthesia is effective in preventing perioperative hypothermia^12^.

In a clinical trial, the authors tested a new technology for patients’ heating. This technology consists of reflective clothing (passive heating, with reduction of body heat loss through a radiation mechanism) that covers the whole body; during the intraoperative moment, reflective clothing can only be used on the upper or lower limbs. In addition, the clothing can be attached to the heated forced air system. The sample consisted of 90 patients randomized into three groups, namely: control group (A) = standard care, without preheating; experimental group 1 (B) = use of reflective clothing for preheating; experimental group 2 (C) = use of reflective clothing for preheating associated with heated forced air system. The preheating time was 30 to 60 minutes. After anesthetic induction, all patients were heated with a heated forced air system. The results showed significantly higher core body temperature in patients of the experimental group 2 (C) during anesthesia and at the end of the surgery. The conclusion is that active preheating showed greater effectiveness in preventing hypothermia^10^.

In this study, the preheating time was 20 minutes. The results showed a statistically significant difference (p = 0.01) between the mean body temperature of the control and experimental group in the T150 period. This difference in body temperature between the groups studied was not evidenced at any other time, and may be related to the temperature and humidity of the operating room air in that specific moment.

There are studies in the literature reporting different preheating times; in some clinical trials where the intervention was effective to maintain body temperature, the preheating time was up to 30 minutes^7^ ^-^ ^8^ ^,^ ^12^. In a recent review of the literature aimed at evaluating the best preheating method and time, the authors stated that the heated forced air system is effective for prevention of perioperative hypothermia. The time of 30 minutes was found to be the suggested average time for preheating, and 10 minutes was the minimum time suggested as significant to reduce hypothermia rates^19^. Results of other studies demonstrated the effectiveness of the intervention with a longer preheating time^6^ ^,^ ^10^.

The environment temperature influences the rate of metabolic heat that is lost from the skin to the environment through radiation, convection and evaporation^17^. Regarding the influence of ambient temperature on body temperature, two studies presented similar results. A prospective cohort study was developed to identify the incidence and magnitude of hypothermia in a heated operating room (26°C) and age-related thermoregulatory response in this circumstance. The participants were divided into groups of age, namely: age between 20 and 40 years, and age between 60 and 75 years. The results showed that heating the operating room had a significant effect in maintaining the body temperature of adult and elderly patients^14^.

A clinical trial involving 791 women undergoing elective caesarean section was conducted to assess whether increased surgical room temperature resulted in decreased neonatal hypothermia and associated morbidities. The authors evaluated 410 infants in the control group and 399 infants in the experimental group. In the control group, the operating room temperature was maintained at 20°C (standard used in the study’s host institution), while in the experimental group, the temperature was adjusted to 23°C. During the intraoperative period, patients were covered with heated cotton bed sheets and received intravenous fluids also heated. If general anesthesia was necessary, the woman would be heated with a heated forced air system and a thermal blanket would be put in the upper part of the body. The babies were wrapped in heated cotton sheets under radiant heat. The rate of neonatal hypothermia was lower in the experimental group (p < 0.001). The body temperature of infants immediately after birth was higher in the experimental group (p < 0.001). At the time of birth, maternal body temperature was lower in the control group (p < 0.001), and this effect persisted until the arrival in the post-anesthetic recovery room (p < 0.001). The authors concluded that a slight increase in the temperature of the operating room reduced the rate of neonatal and maternal hypothermia^15^.

On the other hand, in a clinical trial, in which the effect of preheating the operating room on the body temperature of patients submitted to knee and hip surgery was evaluated, the results indicated that there was no statistically significant difference between the experimental group and the control group in the last measurement performed. The sample consisted of 66 patients, divided into three strata according to BMI, and then randomized into two groups: patients placed in a surgical room with a standard temperature (17°C) and patients placed in a surgical room preheated at 24°C before the patient entered^16^.

In the present study, the two groups presented high mean body temperature (37.9°C) before the application of the intervention (T01), which may have limited heat transfer from the thermal blanket (disposable device of the active system to heat the skin) to the patient’s skin. This assertion is based on a research developed with mannequins whose objective was to determine the effectiveness of heat transfer of the heated forced air system using thermal blankets for the whole body. It was observed that the difference between the temperature of the manikin surface and the temperature in the thermal blanket, called gradient, played an important role in the effectiveness of heating. When the surface temperature of the manikin was 32°C, the transferred heat flow was higher when compared to other surface temperatures (34°C, 36°C and 38°C). The authors concluded that the situation occurred with all heated forced air systems tested, and that heat transfer to an already heated surface is limited^20^.

All participants waited a certain time for the transfer to the operating room to start the surgical anesthetic procedure; this time 42.9 minutes on average in the control group and 38.7 minutes in the experimental group (not statistically significant difference). It is inferred that during the time without active heating some of the heat transferred to the peripheral compartment during preheating may have been lost to the environment by means of convection, radiation and conduction in patients in the experimental group. Thus, the moment without maintenance of preheating may also have contributed to the non-effectiveness of the intervention, because the heat provided by the intervention may have been loss, equaling the groups or even eliminating the preheating effect.

In the analyzed literature, only in two studies^3^ ^,^ ^12^ the authors described the time elapsed between preheating and the beginning of the surgery. In these investigations, the surgery started immediately after preheating, and in only one of them^12^, the results were positive with respect to maintaining patients’ body temperature. In the other studies analyzed, there was no description of the time between preheating and the beginning of the surgery, or of the place where the intervention was conducted^5^ ^-^ ^11^ ^,^ ^13^ ^-^ ^16^.

Regarding the temperature of the system used for preheating, in two studies^3^ ^,^ ^6^, the selected temperature was the same as that of the present research (38°C), and only in one study^6^ the results showed maintenance of patients’ body temperature. In the other studies, the system temperature varied from 42°C to 46°C, with positive ^8^ and negative^7^ ^,^ ^11^ results in body temperature conservation. The authors of the other studies did not present clearly the temperature of the system adopted in the preheating.

The study presented some limitations, namely: the room temperature where the preheating was performed was not measured; blinding, which is advised for clinical trials, was not possible due to the type of equipment used; and the time elapsed between the end of the intervention and the start of the surgery. We recommended therefore for future research the application of preheating inside the operating room, as well as using the heated forced air system at a temperature higher than 38°C (medium power of the equipment).

## Conclusion

The results of the randomized clinical trial showed that preheating with heated forced air system had a similar effect to the usual care in the body temperature of patients undergoing elective gynecological surgeries.
